# Progressive posterior cortical dysfunction

**DOI:** 10.1590/S1980-57642010DN40100013

**Published:** 2010

**Authors:** Fábio Henrique de Gobbi Porto, Gislaine Cristina Lopes Machado, Lilian Schafirovits Morillo, Sonia Maria Dozzi Brucki

**Affiliations:** 1 MD, Behavioral and Cognitive Neurology Unit, Department of Neurology, and Cognitive Disorders Reference Center (CEREDIC); Hospital das Clínicas of the University of São Paulo, São Paulo SP, Brazil.; 2 MD, Department of Radiology, Hospital A.C. Camargo, São Paulo SP, Brazil.; 3 MD, MSc, Behavioral and Cognitive Neurology Unit, Department of Neurology, and Cognitive Disorders Reference Center (CEREDIC); Hospital das Clínicas of the University of São Paulo, School of Medicine, São Paulo SP, Brazil. Discipline of Geriatric Medicine, Hospital das Clínicas of the University of São Paulo, School of Medicine, São Paulo SP, Brazil.; 4 MD, PhD, Neurologist from the Cognitive and Behavioral Neurology Group (University of São Paulo); Psychobiology Department (Federal University of São Paulo); Hospital Santa Marcelina, São Paulo SP, Brazil.

**Keywords:** posterior progressive cortical dysfunction syndrome, Balint’s syndrome, visuospatial dysfunction, visual agnosia

## Abstract

Progressive posterior cortical dysfunction (PPCD) is an insidious syndrome characterized by prominent disorders of higher visual processing. It affects both dorsal (occipito-parietal) and ventral (occipito-temporal) pathways, disturbing visuospatial processing and visual recognition, respectively. We report a case of a 67-year-old woman presenting with progressive impairment of visual functions. Neurologic examination showed agraphia, alexia, hemispatial neglect (left side visual extinction), complete Balint’s syndrome and visual agnosia. Magnetic resonance imaging showed circumscribed atrophy involving the bilateral parieto-occipital regions, slightly more predominant to the right. Our aim was to describe a case of this syndrome, to present a video showing the main abnormalities, and to discuss this unusual presentation of dementia. We believe this article can contribute by improving the recognition of PPCD.

## Abstract (pt)

Disfunção cortical posterior progressiva (DCPP) é um síndrome insidiosa caracterizada por distúrbios do processamento visual superior. Ela afeta tanto a via dorsal (occipito-parietal) como a ventral (occipito-temporal), alterando o processamento visuoespacial e reconhecimento visual, respectivamente. Nós relatamos o caso de uma mulher de 67 anos com dificuldade progressiva nas funções visuais. O exame neurológico mostrou agraphia, alexia, negligência especial (extinção visual à esquerda), síndrome de Balint complete e agnosia visual. A ressonância magnética mostrou atrofia nas regiões parieto-occipitais, mais evidente à direita. Nosso objetivo foi descrever um caso desta síndrome, apresentar um vídeo mostrando as principais anormalidades e discutir esta apresentação incomum de demência. Nós esperamos que o artigo possa facilitar o reconhecimento da DCPP.

Progressive posterior cortical dysfunction (PPCD) is an insidious syndrome characterized by prominent disorders of higher visual processing. It affects both dorsal (occipito-parietal) and ventral (occipito-temporal) pathways, disturbing visuospatial processing and visual recognition, respectively.^1-3^ PPCD may present a combination of partial or complete Balint’s syndrome and Gerstmann’s syndrome (agraphia, acalculia, finger agnosia and right-left side confusion), alexia, transcortical sensory aphasia and visual agnosia. Visuospatial processing disorders are more frequent, indicating a predilection for the dorsal occipito-parietal stream.^1,4^ Our aim was to describe a patient with this syndrome and to present a video showing the main abnormalities.

## Case report

A 67-year-old right-handed woman, with nine years of formal schooling presented at our outpatient memory clinic complaining of progressive forgetfulness, difficulties in spatial orientation and visual acuity. She reported getting lost in familiar places, having difficulty in seeing the objects in front of her, “blurred vision” and problems learning new information. Her son-in-law reported that her topographic disorientation initially occurred in familiar streets but at the time of the consultation she was disoriented even within her own home.

He also complained that she frequently bumped into the furniture as if she was unable to see it and looked for the objects in front of her by feeling for them with her hands (like a blind person). He confirmed her complaint of memory loss. She became progressively dependent, mainly due to the visual deficits.

The patient’s visual impairment was initially attributed by her family to being an ophthalmologic problem, despite an unremarkable previous formal ophthalmologic evaluation. She had hypertension, diabetes and dyslipidemia. She reported no family history of dementia. Cranial nerves, muscular strength, deep tendon reflexes, cerebellar, tonus and sensory examination were unremarkable. Visual field confrontation exam was apparently normal in spite of the evaluation difficulty typically found in these patients. She presented agraphia, alexia, hemispatial neglect (left side visual extinction) and complete Balint’s syndrome^5^ (simultanagnosia - disturbance of the ability to perceive the visual fields as a whole, optic ataxia - impairment of target pointing under visual guidance and ocular apraxia - inability to shift gaze at will towards new visual stimuli). The patient could not recognize a simple object when it was presented in front of her (visual stimuli) but promptly did so when it was placed in her hand or upon hearing its sound – visual agnosia (Video). The cognitive evaluation is shown in Table 1. Laboratory blood screening work up for dementia performed according to national^10^ and international^11^ consensus was unremarkable. Magnetic resonance imaging showed circumscribed atrophy involving the bilateral parieto-occipital regions, slightly more predominant to the right (Figure). The diagnosis of progressive posterior cortical dysfunction syndrome (PPCD) was made based on clinical and imaging grounds.

**Table 1:** Brief Cognitive evaluation.

Test		Score
MMSE^6^	Global cognitive function screening test	10/30
Phonological fluency^7^ (FAS)	Language and executive functions	18
Semantic fluency^8^(animals)	Language and executive functions	4
CAMCOG^9^	Global cognitive function test	22/107

MMSE, Mini Mental State Examination; CAMCOG (part of the CAMDEX interview).

**Figure:**
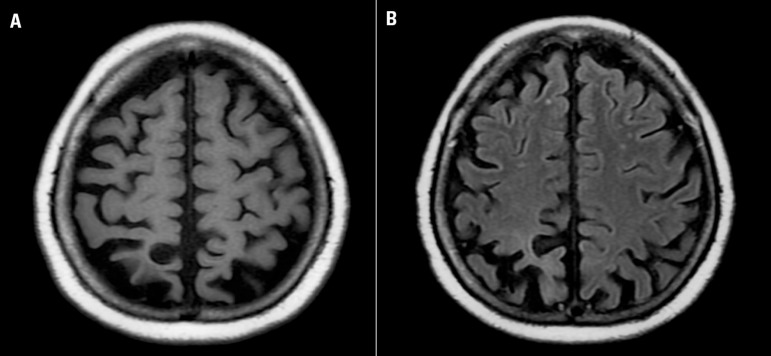
[A] Axial T1WI MR reveals marked occipital and parietal cortical atrophy. [B] Axial T1WI MR shows assimetric atrophy of occipital lobes that predominates in right lobe.

## Discussion

In a multiple case study^4^ of 12 patients with PPCD, only one patient presented visual agnosia, which is a relatively uncommon finding.^1^ Agnosia is often defined as “a normal perception stripped of its meaning”. According to this concept, visual agnosia is characterized by normal visual perception in the absence of the meaning of what was seen, i.e. perception without recognition.^5^ Thus, it is necessary to ensure that the stimuli is introduced in the effective portion of the visual field (normally a small fragment of the visual field that correspond to the macular representation). If this measure is not taken, patients with visual spatial analysis disorders could be misdiagnosed as agnosic.^5^

PPCD is capable of damaging both the dorsal (related to spatial analysis) and ventral (related to spatial recognition) visual processing streams, where both deficits can be found in the same patient.

PPCD is also known as posterior cortical atrophy (PCA)^3,12^ based on predominantly posterior neuroimaging findings and cortical pathologic involvement. Recently, the expression PPCD was suggested^2^ because circumscribed atrophy is not universally found, and PPCD is a descriptive expression rather than as anatomopathologic one. It is essential to rule out primary ophthalmologic causes when diagnosing PPCD.^3^ Some proposed diagnostic criteria have been published^3,13^ (Table 2).

**Table 2:** Proposed diagnostic criteria^3^.

**Core features**
• Insidious onset and gradual progression
• Presentation of visual complaints in the absence of significant primary ocular disease explaining the symptoms
• Relative preservation of anterograde memory and insight early in the disorder
• Disabling visual impairment throughout the disorder
• Absence of stroke or tumor
• Absence of early parkinsonism and hallucinations
**Any of the following findings**
• Simultanagnosia with or without optic ataxia or ocular apraxia
• Constructional dyspraxia
• Visual field defect
• Environmental disorientation
• Any of the elements of Gerstmann syndrome
**Supportive features**
• Alexia
• Presenile onset
• Ideomotor or dressing apraxia
• Prosopagnosia
**Investigations**
• Neuropsychological deficits referable to parietal and/or occipital regions
• Focal or asymmetric atrophy in parietal and/or occipital regions on structural imaging
• Focal or asymmetric hypoperfusion/hypometabolism in parietal and/or occipital regions on functional imaging

According to the concept of the syndrome, PPCD can have several etiologies. Atypical presentation of Alzheimer’s disease, with pathological findings, predominantly in dorsal brain areas and relative sparing of the medial temporal lobe, has been the most frequent type of pathology described in autopsy reports. However, corticobasal degeneration, Lewy body dementia and even Creutzfeldt-Jakob disease are all possible etiologies.^2,3^ A long-term follow-up of these patients and vigilance for new neurologic symptoms and signs are important because the initial presentation can change over time, defining the diagnosis (for example: a new onset unilateral extra-pyramidal rigidity indicating corticobasal degeneration syndrome, bilateral parkinsonism and visual hallucination supporting the diagnosis of Lewy bodies dementia and so forth). It is noteworthy that partial or complete Balint’s syndrome is one of most characteristic disturbances of PPCD although Balint’s syndrome is more often caused by bilateral infarction in watershed areas between the anterior and posterior cerebral artery circulation, causing damage to occipito-parietal cortex. Hemodynamic mechanisms, mainly sudden and severe hypotension, are often involved in the genesis of this type of stroke.

In conclusion, we believe that this article may contribute by improving the recognition and diagnosis of PPCD, especially among general neurologists and physicians. We also emphasize the need for further clinical, neuropsychological, imaging and neuropathological reports to better the understanding of the peculiar pattern of this degenerative disease.
